# Dr. Nottingham’s New Splint

**Published:** 1842-10-08

**Authors:** J. Nottingham

**Affiliations:** Surgeon


*New Splint.* By J. Nottingham, Surgeon.—In this splint the part resembling Mr. Liston's is somewhat modified. It has no screw under the hinge which joins the leg and thigh-pieces, each of which, instead of being kept along with the other at any particular angle by the said screw, is supported on a forked iron rod, attached to it by a hinge-joint underneath; its other extremity being so arranged as to slide in an horizontal groove formed on each side of the support, or frame of the splint, which rests on the bed. This sliding motion is arrested at any point along the frame, by a screw which is attached to the foot of either support, and so the splint at the angle required rendered firm in any position upon its frame.

The sides of the frame are covered with plates of brass, which facilitate the motions required ; and at the fore part, corresponding to the situation of the foot, the splint and frame are so articulated as to prevent any lateral motion of the former upon the latter ; but this juncture can be separated if required, and the whole length of the conjoined portions of the splint raised to any convenient angle with the frame, thus allowing the limb to be placed in that position which is frequently chosen for the treatment of fractured patella ; or the conjoined portions may be laid flat upon the frame, so as to afford a perfect apparatus for the treatment of fracture in the extended position.

An additional thigh-piece slides under the upper or posterior portion of the instrument, and may be drawn out so as to lengthen it, and then fixed by the accompanying screw. For this extremity, also, additional lateral pieces are contrived, external and internal, and each fixed, as circumstances may require, by two screws. These will occasionally be wanted during the treatment in the straight position ; the outside one to pass beyond the crista of the ilium, the other to go towards the upper and inner part of the thigh. The foot board may be gently drawn downwards, or pushed upwards, by the screw attached to it, which is seen projecting from the extremity of the instrument. By this means the position of the foot may be regulated, and any requisite extension easily and securely obtained.

The fundamental part of the contrivance for protecting the heel is the same as in Mr. Liston's splint ; but the leather sock belonging to it affords an additional facility for raising the foot from the iron part of the apparatus, and thus, as it were, slinging it more safely. The rectangular frame of wood, without being heavy, is of such shape and extent as to rest securely on the bed ; and its steadiness is further increased by transverse bars of iron at either end, which fold up when not wanted, so as to take nothing from the portabilityof the instrument, allowing it to be packed in a case of moderate size.

There is a long pillow for the splint, a body girth, and padded strap for the groin, rendering the appendages complete.

The advantages which this apparatus possesses over others previously employed are too obvious to require any particular notice at present.

It serves at once the purposes of a double-inclined plane, and of a straight splint.

It admits of being employed for raising and supporting the leg and thigh, in the treatment of fractured patella.

It combines the advantages of several splints of different sizes.

It affords a more complete protection to the heel than any splint hitherto employed.

It supplies a means of producing extension in cases of fracture, where this may be required, in a most safe and gradual manner, and withal has less the aspect of a complicated apparatus than many contrivances hitherto in use.

It should have been remarked above, that the splint is connected with its frame at the upper extremity, by a common brass hinge. The forked iron rod before mentioned has one branch of its divisions corresponding to each side of the frame of the splint, each branch carrying its screws along with it.—*Lond. Med. Gaz.* Aug. 19, 1842.

